# Comparison of the characteristics at diagnosis and treatment of children with heterozygous familial hypercholesterolaemia (FH) from eight European countries

**DOI:** 10.1016/j.atherosclerosis.2019.11.012

**Published:** 2020-01

**Authors:** Uma Ramaswami, Marta Futema, Martin P. Bogsrud, Kirsten B. Holven, Jeanine Roeters van Lennep, Albert Wiegman, Olivier S. Descamps, Michal Vrablik, Tomas Freiberger, Hans Dieplinger, Susanne Greber-Platzer, Gabriele Hanauer-Mader, Mafalda Bourbon, Euridiki Drogari, Steve E. Humphries

**Affiliations:** a Lysosomal Disorders Unit, Royal Free Hospital, London, UK; b Centre for Heart Muscle Disease, Institute for Cardiovascular Science, University College London, London, UK; c National Advisory Unit on Familial Hypercholesterolemia, Department of Endocrinology, Morbid Obesity and Preventive Medicine, Oslo University Hospital, Oslo, Norway; d Unit for Cardiac and Cardiovascular Genetics, Department of Medical Genetics, Oslo University Hospital, Oslo, Norway; e Department of Nutrition, University of Oslo, Oslo, Norway; f Department of Cardiology and Internal Medicine, Erasmus Medical Center, Rotterdam, the Netherlands; g Department of Pediatrics and Academic Medical Center, Amsterdam, the Netherlands; h Centres Hospitaliers Jolimont, Lipid Clinic, Haine-Saint-Paul, Belgium; i Third Department of Internal Medicine, General University Hospital and First Faculty of Medicine, Charles University, U Nemocnice 1, 128 08, Prague 2, Czech Republic; j Centre for Cardiovascular Surgery and Transplantation, Pekarska 53, 656 91, Brno, Czech Republic; k Medical Faculty, Masaryk University, Brno, Czech Republic; l Institute of Genetic Epidemiology, Department of Genetics and Pharmacology, Medical University of Innsbruck, Schöpfstraße 41, 6020, Innsbruck, Austria; m Division of Pediatric Pulmonology, Allergology and Endocrinology, Department of Pediatrics and Adolescent Medicine, Medical University Vienna, Austria; n FH Registry of the Austrian Atherosclerosis Society, Vienna, Austria; o Cardiovascular Research Group, Research and Development Unit, Department of Health Promotion and Chronic Diseases, National Institute of Health Doutor Ricardo Jorge, Lisbon, Portugal and University of Lisboa, Faculty of Sciences, BioISI - Biosystems & Integrative Sciences Institute, Lisboa, Portugal; p First Department of Pediatrics, National and Kapodistrian University of Athens and Department of Inborn Errors of Metabolism and Inherited Dyslipidemias, “MITERA” Children's Hospital, Athens, Greece; q Centre for Cardiovascular Genetics, Institute for Cardiovascular Science, University College London, London, UK

**Keywords:** Heterozygous familial hypercholesterolaemia, Paediatric FH, LDL-C concentrations, Statin treatment

## Abstract

**Background and aims:**

For children with heterozygous familial hypercholesterolaemia (HeFH), European guidelines recommend consideration of statin therapy by age 8–10 years for those with a low density lipoprotein cholesterol (LDL-C) >3.5 mmol/l, and dietary and lifestyle advice. Here we compare the characteristics and lipid levels in HeFH children from Norway, UK, Netherlands, Belgium, Czech Republic, Austria, Portugal and Greece.

**Methods:**

Fully-anonymized data were analysed at the London centre. Differences in registration and on treatment characteristics were compared by standard statistical tests.

**Results:**

Data was obtained from 3064 children. The median age at diagnosis differed significantly between countries (range 3–11 years) reflecting differences in diagnostic strategies. Mean (SD) LDL-C at diagnosis was 5.70 (±1.4) mmol/l, with 88% having LDL-C>4.0 mmol/l. The proportion of children older than 10 years at follow-up who were receiving statins varied significantly (99% in Greece, 56% in UK), as did the proportion taking Ezetimibe (0% in UK, 78% in Greece). Overall, treatment reduced LDL-C by between 28 and 57%, however, in those >10 years, 23% of on-treatment children still had LDL-C>3.5 mmol/l and 66% of those not on a statin had LDL-C>3.5 mmol/l.

**Conclusions:**

The age of HeFH diagnosis in children varies significantly across 8 countries, as does the proportion of those >10 years being treated with statin and/or ezetimibe. Approximately a quarter of the treated children and almost three quarters of the untreated children older than 10 years still have LDL-C concentrations over 3.5 mmol/l. These data suggest that many children with FH are not receiving the full potential benefit of early identification and appropriate lipid-lowering treatment according to recommendations.

## Highlights

• The age of HeFH diagnosis varies significantly between 8 European countries.
• The proportion of HeFH children being treated varies across 8 European countries.
• A quarter of FH children on statins have LDL-C above the target (>3.5 mmol/L).
• Many FH children are not getting the full benefit of early diagnosis and treatment.

## Graphical abstract

**Figure:**
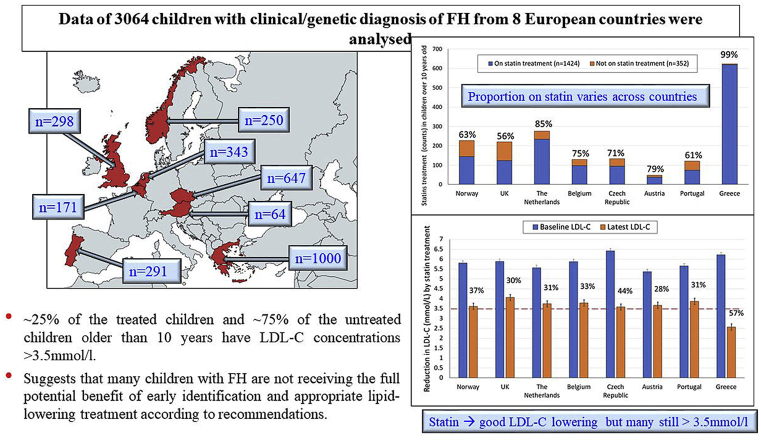

## 1 Introduction

Familial hypercholesterolaemia (FH) is an autosomal dominant inherited disorder characterised by elevated low-density lipoprotein cholesterol (LDL-C) levels from birth [1]. This causes a greatly elevated risk of premature coronary heart disease (CHD) in middle age [2], which can be significantly reduced by statin therapy [1,2]. Recent studies have reported that the prevalence of heterozygous FH (HeFH) is around 1 in 250 in a number of different countries [[3], [4], [5]], though it is currently unknown if this prevalence is the same in all countries in Europe. FH is most often due to carriage of a mutation in the *LDLR* gene, which encodes the low-density lipoprotein receptor (LDL-R), but mutations in apolipoprotein B (*APOB*), and proprotein convertase subtilisin/kexin type 9 (*PCSK9*), can produce a phenotype identical to FH due to mutation in *LDLR* [6]. In patients where no causative mutation can be found a polygenic cause of their hyperlipidaemia is most likely [[7], [8]]. Once identified, subjects with FH can be offered healthy life style advice to decrease their elevated cardiovascular risk (e.g. avoiding or stopping smoking, healthy eating, exercise) and lipid-lowering therapies.

In the last 10 years, many National and European guidelines have been published for the identification and management of children with FH [1,2,[9], [10], [11], [12], [13], [14], [15]]. The UK Simon Broome FH diagnostic thresholds for children under the age of 16 years include: total cholesterol >6.7 mmol/l and LDL-C >4.0 mmol/l [2]. In the UK the 2008 NICE Guideline (CG71) recommends statin therapy should be considered by the age of 10 years [15], while European guidelines on the management of FH in childhood proposed that statin use should be considered from the age of 8 years, and that LDL-C be lowered below 3.5 mmol/l if possible [11]. Both recommend use of Ezetimibe as an adjunct to statin therapy in those over the age of 10 years who are statin-intolerant or who have not achieved the LDL-C target.

The initiation of lipid-lowering therapy in children with FH is determined by factors such as the child's current LDL-C levels, the age of onset of CHD in relatives, and the presence of other CHD risk factors [e.g. obesity or level of Lp(a)] [16]. Although follow-up of children with FH who were started on a statin by the age of 10 years supports the potential CHD benefit [11,17], the age at which statin use should be started, or its intensity to best prevent the onset of adult premature CHD has not been rigorously established, since there are no long-term randomized controlled outcome trials for ethical and practical reasons. There is, however, considerable short term randomized and observational data on the utility of statin therapy in children with HeFH, showing a good safety profile, without liver toxicity side effects, no influence on growth trajectory and excellent efficacy in terms of LDL-C reduction over periods of 2–3 years [[18], [19], [20], [21]].

While the European guidelines and country specific guidelines are relatively similar in their recommendations for the total and LDL-C thresholds for a clinical diagnosis of FH and treatment strategies, adoption of these recommendations are likely to be influenced by local factors such as clinician and parental preferences and the different health care and reimbursement systems for lipid-lowering therapy. The UK National Paediatric FH Register was established in 2012 to collect baseline and long term follow-up data on children with a clinical diagnosis of HeFH in UK [22,23]. In 2017 we obtained funding from the International Atherosclerosis Society (IAS) to collect similar data from seven other European countries, to establish an International Paediatric FH Register and to compare across Europe the characteristics at diagnosis, including the proportion with an identified mutation and the proportion of children with LDL-C < 4.0 mmol/l, and the age of initiation and lipid-lowering effect of statin treatment in the different countries. Although information on children in the UK [22], Portugal [24], The Netherlands [25] and Norway [26] cohorts has been published previously, the novelty of this present study is the analysis of the between-country similarities and differences in diagnostic and treatment strategies currently being used.

## 2 Patients and methods

### 2.1 Register criteria

Based on the UK register [22,23] a “minimum data set” and data dictionary of 86 key variables was developed (available on request from authors). As no funding was available for study-specific (or registry specific) data collection, only centres already having available such data from children and young people (under the age of 18 years) were asked to participate. Any child with a local diagnosis of heterozygous FH (either clinical or genetically verified) were included. Homozygous FH was excluded. For Greece, the referring clinician requested that only those with an identified mutation should be included. This study is therefore a retrospective dataset designed to allow comparison of the different ways in which children with FH are being identified and treated. See Supplementary Table 1 for presentation of the key selection criteria and time frame variables.

### 2.2 Country-specific patient identification

**Norway:** Data were collected retrospectively to a treatment quality-register, from medical records of children below 18 years with a diagnosis of heterozygous FH, visiting the Lipid Clinic, Oslo University hospital during 2014–2016. Only children with a confirmed pathogenic mutation in the *LDLR* gene, the p.R3500Q mutation in *APOB*, or *PCSK9*, or children with LDL-C concentrations >4.9 mmol/l and a first or second degree relative with such a mutation, were included. All genetic tests were performed by the Unit for Cardiac and Cardiovascular Genetics at Oslo University Hospital. Data on diagnosis, lipid levels, other relevant blood chemistry, lipid-lowering therapy, diet and smoking habits were collected. Details has been described before [26]. The treatment quality register was approved by the Regional Committee for Medical and Health Research Ethics and the Data Protection Official at Oslo University Hospital. Informed consent is not required in Norway for this type of data collection used for quality of treatment purposes. Sending fully-anonymized data to UK was approved by the hospital and did not require new Ethics committee approval.

**UK:** All lipid clinics in the UK and paediatricians with an interest in lipid disorders were contacted electronically and details of the register provided. An electronic web based data capture tool was developed to collect information. The register captures routine clinical data, demography, family history, treatments and lifestyle details, and clinicians are sent an electronic reminder to fill in annual follow up data. Full details of the establishment and governance of the Register have been published [22]. Data on children registered between July 2012 to November 2014 were included. Children were diagnosed as having FH based on the UK Simon Broome criteria [2], with the majority having been identified by family studies from an index case with a clinical diagnosis of FH.

**The Netherlands:** Data was collected from all consecutive children with FH who visited the outpatient lipid clinics of the Erasmus MC or Sophia Children Hospital The Netherlands for the first time under 18 years old, between April 1993 and February 2018 and was entered in a database. The diagnosis of FH was based either on identification of a FH pathogenic variant in *LDLR//APOB/PCSK9* or Dutch Lipid Clinic Network criteria with definite FH score ≥8 [25,27]. Children with homozygous FH were excluded. Most children were referred because they had a parent diagnosed with FH. The Medical Ethical Review Committee of the Erasmus MC, The Netherlands, considered the protocol non-Medical Research Involving Human Subjects Act (WMO) therefore review of the protocol was waved (MEC-2017-1197).

**Belgium:** Data were obtained retrospectively from a review of the medical record of one lipid clinic in one department of internal medicine for the vast majority, as well as from a paediatric consultation for a dozen patients over the period 2014–2018. This situation reflects the general situations in Belgium where testing cholesterol levels in children is still rare in paediatric consultation. The children seen in an internal medicine consultation are most often those of parents who are followed in the adult consultations for their FH. The diagnosis of FH in their parent was based either on identification of a FH pathogenic variant in *LDLR//APOB/PCSK9* or Dutch Lipid Clinic Network criteria with definite FH score ≥8 (see above). A small proportion of children were sent directly by their doctor, who have measured lipid concentrations following the discovery of a suspicion of FH or following the start of treatment for acne. FH in children was confirmed using either the LDL-C cut-off previously published [11] or by genetic testing. Collecting the data, encoding in a anonymized file and sending the data to UK was approved by the local hospital ethical committee. Informed consent is not required in Belgium for this type of data collection.

**Czech Republic:** Data were obtained from the Czech MedPed registry, with collection of children data over the period 1998–2018. There are two different ways that children with FH get registered within a national MedPed database. Approximately 50% have been identified through cascade screening. These are children of FH adult patients being invited to visit paediatric FH centre affiliated with a regional adult FH centre. Plasma lipid levels are examined and clinical diagnosis is established. Genetic testing is offered to be done in a child, if a disease-causing mutation is known in the family. The remainder have been identified from a nationally adopted selective FH screening programme or from the other health care-related blood testing. The Paediatric care network is well established in the Czech Republic and 98% of the children receive bi-annual preventive check-ups. Since 1998 paediatricians have been instructed by local guidelines to perform selective dyslipidaemia screening in families affected with a) known familial dyslipidaemia running in the pedigree (e.g. diagnosis established in first/second degree relatives) or b) premature atherothrombotic vascular complications in first/second degree relatives. The paediatrician should ask about the presence of the two for the first time during the preventive check-up at age of 5 and repeat the investigation once again at the check-up at age of 13. Once the response to any of the two questions from the parent/guardian is positive, the child is referred for blood sampling and plasma lipid levels are assessed. Where they exceed age and gender specific values of 95th percentile of total and/or LDL-cholesterol distribution, they are referred to the regional paediatric FH centre for specialised counselling. Here plasma lipid levels are measured again, a thorough medical examination focusing on subclinical atherosclerosis is performed and secondary causes of dyslipidaemia excluded. The suspected FH child's data are then entered in the national database and FH criteria fulfilment is checked by one of two members of the MedPed CZ project who then approve (or disprove) submitting the patients' material for genetic analysis. Genetic testing is offered to the family together with examination of all available relatives of the proband in the case a disease-causing mutation being detected. Patients with FH diagnosis confirmed based on clinical and/or molecular criteria continue being followed at the MedPed centre with a frequency twice a year at minimum.

**Austria:** Data were obtained from an FH registry project initiated by the Austrian Atherosclerosis Society in 2015. This project started as a pilot project at the three Medical Universities in Vienna, Graz and Innsbruck and now also involves other medical centres/hospitals in Austria. By the summer of 2018 (when the data from FH-affected children were evaluated), 350 FH patients had been recruited into the registry. FH-affected children (<19 years old) were (clinically) diagnosed according to the Simon-Broome criteria as described [28]. The software platform Askimed developed at the Institute of Genetic Epidemiology of the Medical University of Innsbruck was used for data entry, management and monitoring (www.askimed.com).

**Portugal:** Data from 294 FH children was collected anonymized from the Portuguese FH Study, a nation-wide study started in 1999 at the National Institute of Health. All children with ages up 21 years old in 2018 referred to this study as index fulfilled FH clinical criteria (Simon Broome) were included and also children that were relatives of adult patients with a causative mutation. All clinicians were contacted to update the information in the last visit on lipid profile, treatment, lifestyle as smoking habits and age of menarche. Updated information was only available for 125/294 individuals.

T**he Greek Paediatric FH Register:** In 1993, the Paediatric FH Registry was started at the Unit for Inherited Metabolic Disorders (IEM) (director Professor Euridiki Drogari) at the Choremio Research Institute, of the 1st Department of Paediatrics, National and Kapodistrian University of Athens, at the “Agia Sofia” Children's Hospital in Athens. The collection period is thus 1993–2018. Paediatricians throughout Greece were requested to measure cholesterol levels in all children around the age of 3 years, and if levels were above the 97th centile for age and sex, the children were referred to the Athens Metabolic Clinic. During the first two visits cascade screening for three continuous generations was performed in all members of the families. Children and adults who fulfilled the Simon Broome clinical and biochemical criteria for FH were offered molecular analysis from University lab dedicated to this purpose. The patients were screened for mutations in three genes (*LDLR/APOB/PCSK9*), and to date, no *APOB* and *PCSK9* mutations have been found in the Greek population [29], and from a database of several thousand cases all 1000 children selected had an identified *LDLR* mutation. When children reached the age of 8 years they start treatment with statins or ezetimibe alone or in combination. Close follow up between three and six months for the lipid profile together with the growth and development during treatment was performed for each child until the age of 17–18 years. The FH adults were referred to Lipid Adult Specialists.

**Approvals:** Approvals of data collection and sharing was obtained in each country according to national regulations. Although data was already fully-anonymized, data was sent as an excel sheet in a password-protected file, with the password sent separately. Data was stored in the UCL Data Safe Haven, which is fully GDPR-compliant.

**Statistical methods:** Results for continuous variables are presented as mean (±standard deviation) and median (with interquartile range), and differences by sex and statin use are tested using Mann-Whitney U tests. Differences in the fall in LDL-C by statin use are adjusted for age using analysis of covariance. Changes in lipid levels are the difference between the baseline registration and follow-up of the patient. Categorical variables are presented as percentages and numbers, and tested using chi-squared tests or Fisher's exact test. Changes in LDL-C by statin use were analysed using analysis of covariance with adjustment for age and length of follow-up. In order to address potential issues of the large sample of children from Greece inflating statistical differences, *p* values for contrasts are presented with and without the inclusion of the Greek children. For conversion to mg/dl, mmol/l concentrations of total and LDL-C should be multiplied by 38.67. In a proportion of Portuguese (6%) children the baseline untreated LDL-C was not available therefore the untreated concentrations were imputed from latest recorded LDL-C using the method as described [30], which adjusts for the type and dose of the lipid-lowering treatment.

## 3 Results

### 3.1 Baseline characteristics

In all countries, the children were identified mainly by cascade screening, except in Portugal and Czech Republic where >80% and 50% respectively of the children were referred as index cases identified from routine health screening tests. Baseline data shown in Table 1 includes 3064 HeFH children (48% boys), with a baseline mean (SD) LDL-cholesterol (LDL-C) of 5.70(1.44) mmol/L. Untreated LDL-C concentrations were ranging from 4.87 mmol/l in Austria to 6.21 mmol/l in Greece (Supplementary Figure 1). The median (interquartile range) age at diagnosis differed significantly, ranging from 3 [1] years in Greece to 11 [6] years in the Netherlands and Belgium. The prevalence of a family history of early CHD in any relative differed significantly (*p* < 0.0001) being higher in countries in the North of Europe than in the South. After excluding the Greek cohort, where all children had an identified FH mutation, the average proportion of the children carrying an identified FH-causing mutation was 79%, ranging from 61% in Portugal to over 90% in Norway, the Netherlands and Belgium. The majority of mutation carriers had a pathogenic variant in the *LDLR* gene. On average 10% of the genetic causes were due to a mutation in *APOB*, though the frequency of the *APOB* defect varied between the countries, being the most common in children from Czech Republic, accounting for 39% of all FH-causing mutations, and not present in the Greek children (Supplementary Table 3).

**Table 1:** Baseline and follow-up characteristics of FH children by country.

	Norway (n = 250)	UK (n = 298)	The Netherlands (n = 343)	Belgium (n = 171)	Czech Republic (n = 647)	Austria (n = 64)	Portugal (n = 291)	Greece (n = 1000)	All excluding Greece (n = 2064)	All (n = 3064)	*p* for overall difference excluding Greece	*p* for overall difference
Median age at diagnosis (IQR) (years)	9 (4)	10 (6)	11 (6)	11 (6)	10 (6)	8 (7)	10 (5)	3 (1)	10 (6)	7 (9)	4x10^−14^	<2x10^−16^
N of males (%)	122 (49)	153 (51)	162 (47)	72 (42)	297 (46)	30 (47)	131 (45)	505 (51)	967 (47)	1472 (48)	NS	NS
N with family history of CHD (%)^a^	NA	62 (21)	56 (16)	38 (24)	53 (8)	17 (27)	44 (15)	NA	270 (13)	270 (15)	<0.0001	<0.0001
N with identified mutation (%)^b^	248 (99)	184 (67)	326 (97)	120 (91)	519 (85)	48 (75)	178 (61)	1000 (100)	1623 (79)	2623 (87)	<2x10^−16^	<2x10^−16^
Earliest TC (mmol/l)	7.26 (1.39)	7.45 (1.51)	7.02 (1.56)	7.41 (1.48)	7.48 (1.49)	6.76 (1.74)	7.23 (1.55)	8.13 (1.22)	7.31 (1.52)	7.58 (1.48)	<2x10^−16^	<0.0001
Earliest LDL-C (mmol/l)	5.35 (1.34)	5.51 (1.49)	5.30 (1.50)	5.51 (1.41)	5.63 (1.44)	4.87 (1.61)	5.30 (1.46)^c^	6.21 (1.25)	5.44 (1.46)	5.70 (1.44)	0.007	<0.0001
Number (%) with LDL-C < 4.0 mmol/l	36 (14.4)	37 (12.4)	55 (16.0)	18 (10.5)	42 (6.5)	18 (28.1)	23 (7.9)	6 (0.6)	229 (11.1)	235 (7.7)	3.5x10^−8^	<2x10^−16^
Earliest TG (mmol/l)	0.93 (0.48)	1.04 (0.54)	1.00 (0.53)	1.06 (0.65)	1.03 (0.56)	0.95 (0.52)	1.00 (0.55)	0.83 (0.39)	1.01 (0.55)	0.95 (0.51)	NS	<0.0001
Earliest HDL-C (mmol/l)	1.46 (0.36)	1.40 (0.33)	1.34 (0.42)	1.43 (0.38)	1.39 (0.39)	1.39 (0.35)	1.46 (0.40)	1.51 (0.29)	1.41 (0.38)	1.44 (0.36)	0.0002	<0.0001
N on statin treatment (%)^a^	145 (58)	134 (45)	253 (74)	117 (68)	98 (16)	44 (69)	77 (27)	834 (83)	868 (42)	1702 (56)	<2x10^−16^	<2x10^−16^
N of children with follow-up data (%)	245 (98)	293 (98)	309 (90)	162 (95)	190 (29)	63 (98)	139 (48)	1000 (100)	1401 (68)	2401 (78)	<2x10^−16^	<2x10^−16^
Median length follow up (IQR) (yrs)	5 (8)	1 (5)	5 (20)	1 (8)	5 (24)	3 (8)	4 (9)	8 (10)	4(6)	6 (7)	<2x10^−16^	<2x10^−16^
Only those on statin treatment												
Latest TC (mmol/l)	5.32 (1.25)	5.82 (1.36)	5.30 (1.36)	5.79 (1.46)	5.33 (1.26)	5.49 (1.40)	5.67 (1.27)	4.22 (0.29)	5.50 (1.35)	4.86 (1.17)	<0.0001	<2x10^−16^
Latest LDL-C (mmol/l)	3.61 (1.22)	4.06 (1.37)	3.74 (1.29)	3.79 (1.34)	3.58 (1.19)	3.67 (1.35)	3.87 (1.29)	2.57 (0.33)	3.76 (1.29)	3.17 (1.11)	0.04	<0.0001
Latest TG (mmol/l)	1.31 (0.23)	0.96 (0.43)	0.98 (0.69)	1.14 (0.67)	0.98 (0.59)	0.95 (0.51)	0.99 (0.49)	0.66 (0.26)	1.05 (0.57)	0.85 (0.48)	NS	0.05
Latest HDL-C (mmol/l)	1.34 (0.39)	1.39 (0.30)	1.37 (0.34)	1.45 (0.39)	1.32 (0.30)	1.43 (0.36)	1.32 (0.29)	1.63 (0.28)	1.37 (0.34)	1.50 (0.34)	0.05	0.02
Reduction in LDL-C by statins (mmol/l)	2.20 (1.26)	1.85 (1.43)	1.87 (1.60)	2.08 (1.81)	2.88 (1.34)	1.70 (1.91)	1.83 (1.34)	3.65 (1.23)	2.06 (1.55)	2.86 (1.61)	7.8x10^−9^	<2x10^−16^
% reduction in LDL-C by treatment	36.9	30	30.9	32.7	43.9	28.1	31	57.4	33.5	46	<0.0001	<2x10^−16^
N with LDL-C>3.5 mmol/l (%)	60 (41)	70 (52)	111 (44)	64 (55)	43 (44)	20 (46)	40 (52)	5 (1)	408 (47)	413 (24)	0.04	<2x10^−16^
N of >10 year olds with LDL>3.5 mmol/l (%)	59 (41)	61 (56)	101 (44)	50 (52)	42 (46)	16 (42)	38 (52)	5 (1)	367 (46)	372 (23)	NS	<2x10^−16^
N achieved 50% LDL-C reduction by statins (%)	37 (26)	20 (15)	59 (23)	31 (27)	36 (37)	9 (21)	12 (16)	705 (85)	204 (24)	909 (53)	0.02	<2x10^−16^
N of >10 year olds with 50% LDL-C reduction (%)	37 (26)	20 (16)	59 (25)	27 (28)	34 (36)	9 (24)	12 (16)	526 (85)	198 (25)	724 (51)	NS	<2x10^−16^

Continuous variables are presented as mean (±standard deviation) and median (with interquartile range).

NA = not available, NS = not significant.

a NAs excluded from %.

b Calculated on those with DNA test done.

c Proportion corrected for treatment.

**Fig. 1:**
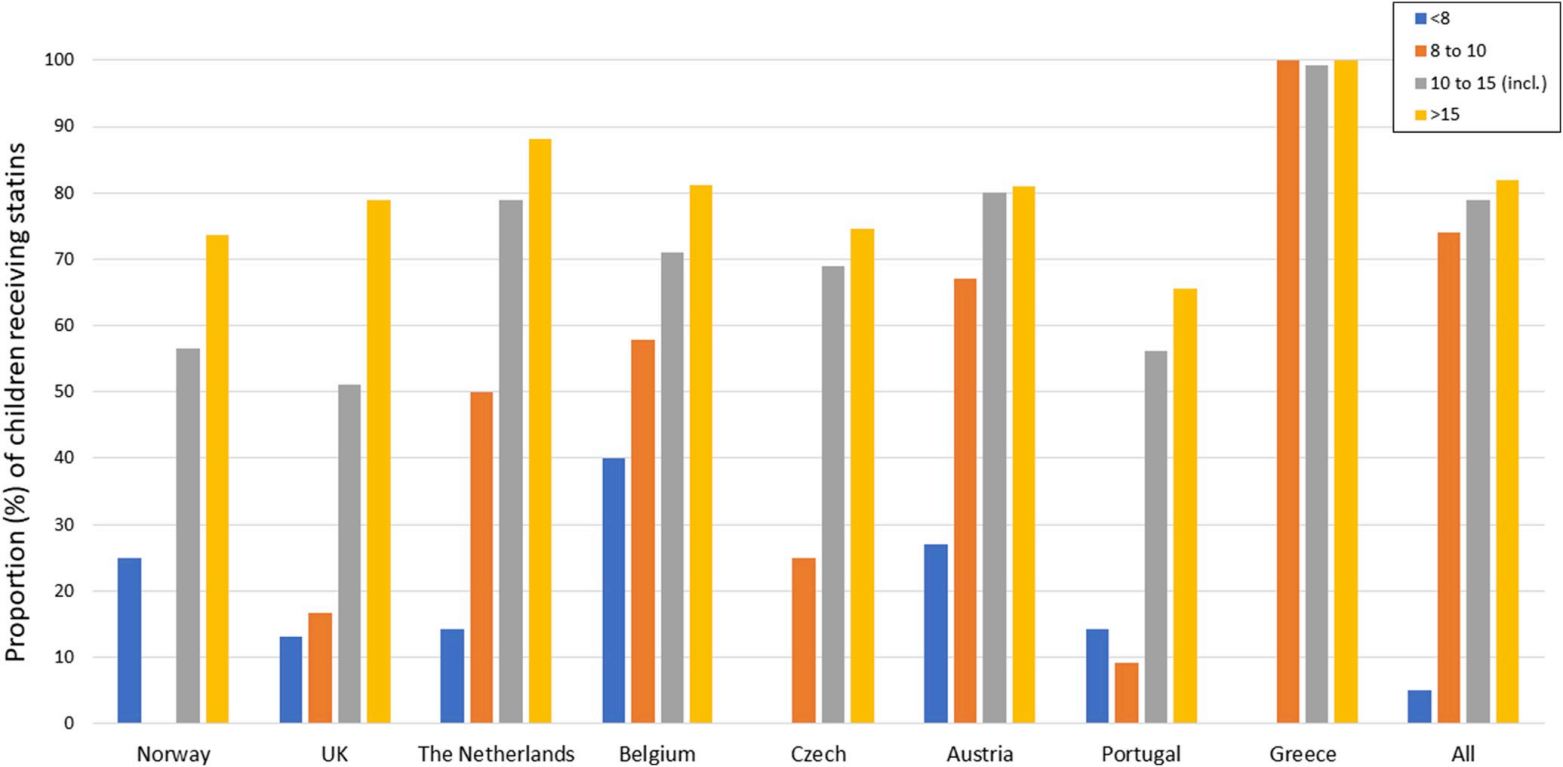
Proportions of children receiving statins by age at follow up per country. Children with follow-up data were grouped into age category (1. younger than 8 years, 2. from 8 to 10 years, 3. 10–15 years, and 4. over 15 years of age).

Overall, more than 90% of the children had an untreated LDL-C of >4.0 mmol/l, with the lowest proportion being 71.9% in Austria and the highest 99.4% in Greece. The overall characteristics of the children with LDL-C below 4.0 mmol/l are presented in Supplementary Table 2. Compared to those with baseline LDL-C >4.0 mmol/l the <4.0 mmol/l group had a marginally higher proportion with a reported family history of CHD (18.9% *vs.* 14.5%, *p* = 0.06) and as expected lower mean total- and LDL-C and lower triglycerides, with fewer of these children receiving a statin (23% *vs.* 59%, *p* < 2x10^−16^). The proportion with a FH-causing mutation was not different between the two groups (87.9% *vs.* 89.3%, *p* = NS).

### 3.2 Lipid-lowering therapy

Follow-up data was available for over 90% of children, although with less data from the Czech and Portuguese cohorts with 29% and 48%, respectively. The median (interquartile range) follow-up period was 6(7) years. Over this period, a considerable proportion of the children were initiated on lipid-lowering therapy. As shown in Supplementary Table 4, the commonly used statins were Atorvastatin (47%; n = 794), Simvastatin (32%, n = 537), Rosuvastatin (13%, n = 233), and Pravastatin (8%, n = 131). Of all those patients receiving any form of a treatment (n = 1789) a small proportion were on resins (2%, n = 36). There was a significant difference in the proportion of children taking Ezetimibe between countries, ranging from 0% in the UK to 78% in Greece (Supplementary Table 4). Overall, 46% of those on treatment were receiving Ezetimibe, mostly (99%) as a combination treatment with a statin. No patients were on fibrates and the use of plant stanols was limited (0.1%, n = 1).

**Fig. 2:**
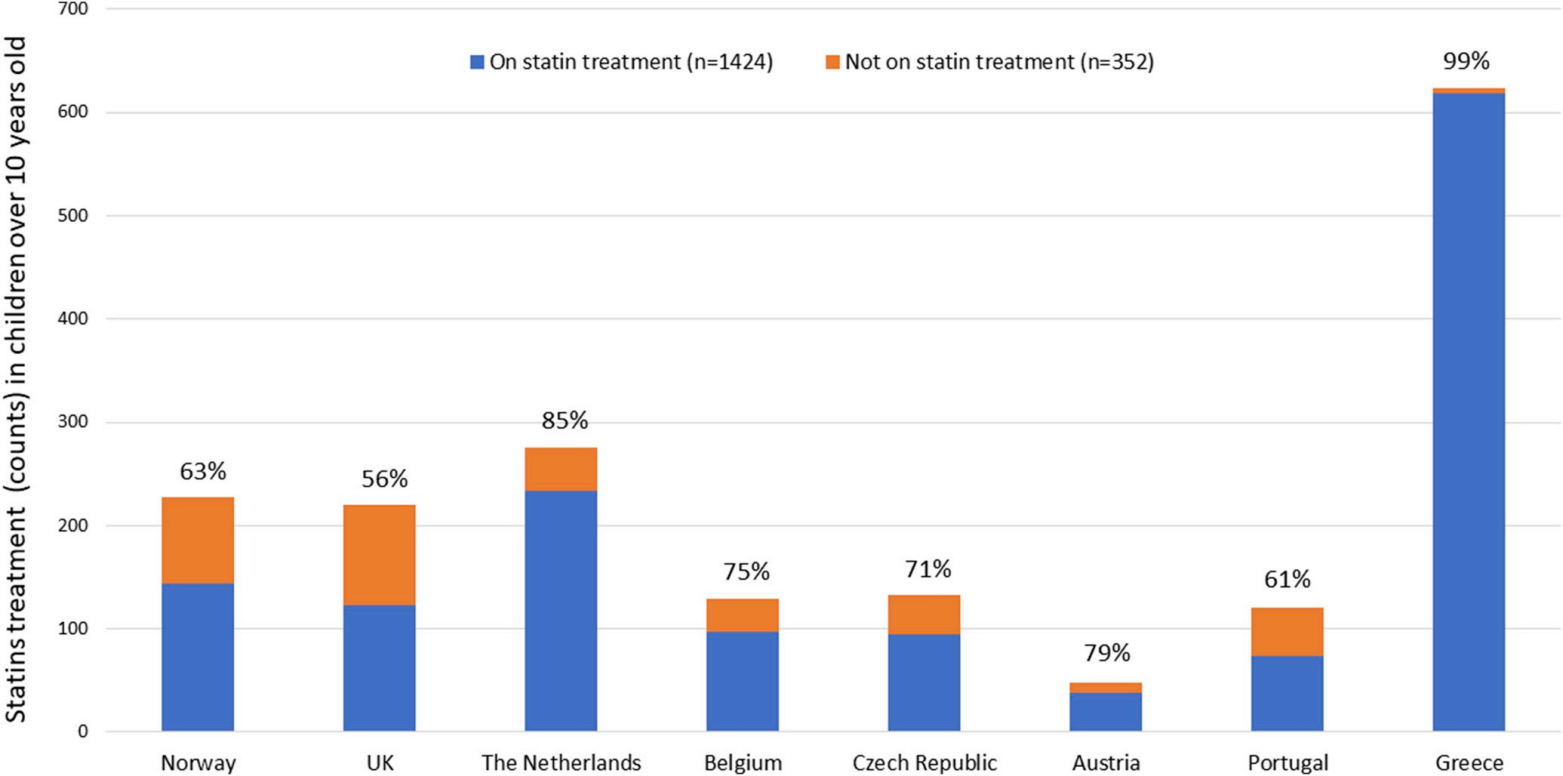
Statin treatment in children older than 10 years of age (at follow-up) per country. Stacked bars represent number of treated and untreated children in each cohort. The percentage on top of each bar shows the proportion of children on statins.

The proportion of children taking lipid lowering therapy by follow-up age is shown in Fig. 1 (and Supplementary Table 5) and the baseline characteristics of those later taking and not taking a statin are shown in Supplementary Table 6. Overall, there was a significantly higher proportion of those where a mutation had been identified in the treated compared to the not treated group (93% *vs.* 76%, *p* < 2.2 x 10^−16^) and a slightly higher proportion of boys than girls on treatment (50% *vs.* 46% *p* = 0.02). As shown in Supplementary Table 8, possible factors explaining this are that a higher proportion of boys than girls had a detected mutation (87% *vs.* 84% *p* = 0.05), and a family history of CHD (10% *vs.* 8% *p* = 0.01), but overall the boys had a lower mean age (7yrs *vs.* 8yrs (*p* = 0.0002) and a lower mean baseline total cholesterol (7.48 mmol/l *vs.* 7.62 mmol/l, *p* = 0.01). At diagnosis, the mean concentrations of triglycerides were slightly lower (*p* = 0.008) and HDL were slightly higher (0.02) in those who were subsequently on statin treatment. Mean diagnostic levels of total and LDL-C were significantly higher in the subsequently treated group (for LDL-C, mean (SD) 6.01 (±1.35) mmol/l *vs.* 5.26 (±1.43) mmol/l, *p* < 2.2 x 10^−16^). The number of children under the age of 8 with available follow-up data was small, representing only 11% of all cohorts (n = 256), (7% when the Greek cohort was excluded). Nevertheless, as expected, in all countries, the proportion of children on a statin under the age of 8 years was low, being overall 5% and ranging from 0% in Czech Republic and Greece to 40% in Belgium. The proportion taking a statin increased with increasing age, and overall was 74% in 8–10 year olds, 79% in 11–15 year olds and 82% in those over the age of 15 years. As shown in Fig. 2, significant between-country differences were apparent, with the proportion of children aged over 10 years not taking statins ranging from 1% in Greece to 44% in the UK (Chi^2^ = 270, df = 7, *p* < 2.2 x 10^−16^).

**Fig. 3:**
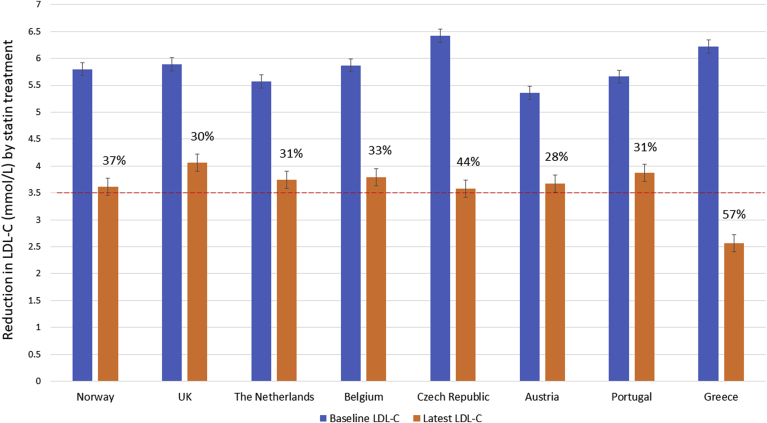
Baseline and treated LDL-C in children who went on receiving statins. The percentage on top of latest LDL-C bars represent the reduction in LDL-C by treatment in each cohort.

As shown in Table 1 and Fig. 3, in those on statin treatment, LDL-C levels were significantly reduced compared to the values at the time of FH diagnosis by an average of 46% (2.86 (1.61)mmol/l), with children in Greece achieving a 57.4% reduction. There were minor differences between the rest of the countries in the extent of LDL-C lowering seen (ranging from 28.1% in Austria to 43.9% in Czech Republic). At follow-up, in children older than 10 years, LDL-C was significantly lower in those receiving the treatment (3.20 (1.1) *vs.* 4.32 (1.3), *p* < 2.2 x 10^−16^), as shown in Supplementary Table 9. Treatment reduced LDL-C levels below the recommended 3.5 mmol/L cut-off in 77% of over 10 year olds (in 55% if the Greek cohort was excluded). However, of those over 10 years of age, who did not receive treatment, 66% had LDL-C > 3.5 mmol/L at the latest visit (Supplementary Table 9). Of the 352 children over 10 years old not on a statin, 66% had levels over the suggested target of 3.5 mmol/l. Overall, of the children over the age of 10 years on statins, 42% were also taking ezetimibe (Supplementary Table 4). As expected the mean (±SD) baseline LDL-C of those taking ezetimibe was higher than in those not on ezetimibe (6.56 (±1.29) mmol/l *vs.* 5.57 (±1.32) mmol/l, *p* < 2.2 x 10^−16^) and the treated LDL-C was lower (2.73 (±0.66) *vs.* 3.56 (±1.26) mmol/l, *p* < 2.2 x 10^−16^). The overall mean (SD) reduction in LDL-C in those taking ezetimibe was higher (56.9% *vs.* 35.4%, *p* < 2.2 x 10^−16^) and the proportion achieving a treated LDL-C below 3.5 mmol/l was higher (92% *vs.* 53%, *p* < 2.2 x 10^−16^).

### 3.3 Treatment in those >10 years

Finally, we examined in each country data the characteristics of the children over the age of 10 years, which is the age by which the UK NICE FH guideline [15] and the European Consensus guidelines [11] recommend that initiation of statin therapy should be considered. Austria was excluded from the analysis as no follow-up data was available. As shown in Fig. 2 and Supplementary Table 9 the proportion of the children receiving statin therapy varied significantly, ranging from 56% in the UK to 99% in Greece. In those not being treated with a statin at follow-up, the mean TC and LDL-C concentrations recorded at time of diagnosis and referral (baseline) were significantly lower than those on statin treatment on follow-up, and for example, overall, in the children over 10 years not on statins, LDL-C concentrations at diagnosis were 20% lower than those who went on to receive treatment (4.85 (±1.3) *vs*. 5.99 (±1.4) mmol/l, *p* < 2.2 x 10^−16^). In addition, the proportion of those who were on statins who had an identified FH-causing mutation was significantly higher than in those who were not on statins (92% *vs.* 70%, *p* < 2.2 x 10^−16^).

## 4 Discussion

This analysis of one of the biggest sets of data of children with FH examined to date, with 2623 with a known mutation, has made three major findings. The first is that across the eight European countries the mean age at diagnosis is very different, ranging from 3 years in Greece to 11 years in the Netherlands and Belgium. This is not surprising given the very different care-pathways, policies and diagnostic strategies used in the different countries and to a large extent reflects the maturity of the FH child diagnostic work across Europe, with the paediatrician in Greece having started clinical practice more than 20 years ago, routinely testing cholesterol concentrations in all children before the age of 3. In the other countries where diagnosis is performed mainly through family cascade screening (after known diagnosis in parent) the median age at diagnosis is between 8 and 11 years, which is in line with paediatric FH guidelines that recommend the testing and identification of children at risk of FH by the age of 8–10 years [[9], [10], [11], [12], [13], [14]]. The lipid profile at diagnosis is relatively uniform across countries, with mean LDL-C 5.70 (±1.44) mmol/l, although with Greece having the highest and Austria the lowest values, due most probably to patient selection criteria. Of the children, more than 88.5% had an untreated LDL-C of >4.0 mmol/l, which is the diagnostic cut-off recommended by the UK Simon Broome Register. In all countries, triglyceride values were low. Data on the family history of CHD was not collected in all countries, but showed a modest north-south gradient, as has been reported for the general population. The proportion of children with an identified mutation varied significantly across countries, but this mainly reflects the availability of DNA testing services. The cohort from Greece was selected from a large data base and only those with an identified mutation were included. Previous work has identified a mutation in 53% of the children on the Greek database [31]. To date no patient in Greece has been identified carrying an *APOB* or *PCSK9* mutation and 6 *LDLR* mutations together explain ~80% of patients with a detectable mutation [29]. As expected from previous country comparisons on adult patients with FH, the proportion of children with *LDLR, APOB* or *PCSK9* mutations also varied significantly between the countries, and analysis of the relationship between the identified mutation and patient characteristics will be presented elsewhere.

The second major finding relates to the proportion of children taking a statin, the different ages where statin therapy has been initiated, and the different statins being used. In line with published recommendations and licensing requirements, in all countries the proportion of children on a statin under the age of 8 years was low, but with wide country differences ranging from 0% in Czech Republic and Greece to 40% in Belgium. These proportions need, however, be taken with caution as the number of children under 8 years old with follow-up data was small, accounting for only 7% of the cohort (n = 94), when excluding Greece. The proportion taking a statin increased with increasing age, and by the age of 15 years 79% of children were taking a statin, but again with large between-country differences, with the proportion not taking statins at the age of 15 ranging from 1% in Greece to 49% in the UK. Overall, a slightly higher proportion of boys than girls were on a statin at follow-up, and although this difference may at least partly be explained by the higher proportion of boys than girls with a detected mutation and a higher prevalence of a family history of CHD, baseline levels of total cholesterol were lower in boys than girls, it does suggest that girls may be being undertreated. While Atorvastatin is the most common statin and is used in all countries, simvastatin is also commonly used in all countries except Norway and rarely in Belgium, while pravastatin is only used commonly in UK, The Netherlands, Belgium and Portugal.

As expected, statin treatment lowered mean LDL-C levels substantially, with children in Greece achieving a 57.4% reduction and minor differences between the rest of the countries, ranging from 28% in Austria to 44% in the Czech Republic. In part, the large LDL-C reduction in children in Greece is a result of them having the highest LDL-C at diagnosis, but of note, 78% of the Greek children were also taking ezetimibe. This may also in part be because of healthy lifestyle and dietary advice being given from an early age. Differences in LDL-C lowering are explained mostly because of the different mix of statin used in the different countries. As the only licensed hydrophilic statin for children under 10 years of age, Pravastatin was being taken by between one quarter and one third of children in UK, Netherlands and Portugal, but by few or no children in the other countries. A high potency statin (atorvastatin or rosuvastatin) was being taken by essentially all children in Norway and Belgium, but by 41% of children in Portugal, and between 51% and 73% in the other countries.

Use of ezetimibe as an adjunct to statin therapy is recommended for adults with FH who are statin intolerant or who fail to reach target on statin alone [1,15] and for children over the age of 10 years [11,15], where efficacy and safety have been documented [32]. Apart from in Greece, ezetimibe was used in all countries at a low and varying frequency as an adjunct to statin therapy. This low use might have been caused by the relatively high price at the time of analysis and because of relatively limited evidence of its use in children. However, the data here shows that, as expected, ezetimibe use lowers LDL-C significantly and that more than 90% of children taking a statin plus ezetimibe achieve LDL-C below the EAS guideline recommendation of 3.5 mmol/l [11], compared to only 53% of those on statin only. Overall, 23% of the treated children older than 10 years still had LDL-C values above the EAS recommendation of 3.5 mmol/l, and apart from the Greek children (where 99% achieved this target), between 41 and 56% of treated children had LDL-C above this level. Poor adherence (for example during adolescence) or scepticism among doctors or parents to increase statin dose or prescribe additional agents may be a contributing factor, but we did not collect data on this.

The third major finding is that a significant proportion of the children above the age of 10 years who were not on lipid-lowering therapy had LDL-C concentrations above the 3.5 mmol/l EAS recommendation for statin initiation). In the dataset as a whole, 352 (20%) of the 1776 children who were over the age of 10 years were not on a statin. Mean latest LDL-C for these untreated children was 4.32 (1.33) mmol/l and 233 (66%) had LDL-C over 3.5 mmol/l. There may be a number of reasons why a particular child is not taking lipid-lowering therapy, but we were unable to collect any standardised information about this. In the UK register, reasons for not being on lipid-lowering medication included weak or absent evidence of a family history of early CHD, which would support the decision to delay initiation, and parental concerns about safety, (particularly if the affected parent had experienced statin-related side effects). Some UK clinicians were also waiting to receive DNA testing results before statin initiation [22]. It is likely that such issues are also seen in all European countries.

A small proportion (<10%) of the children had an untreated LDL-C below the Simon Broome diagnostic threshold of 4.0 mmol/l. Perhaps unsurprisingly, fewer of these children were receiving a statin (23% vs 59%), since for the majority their LDL-C is below the threshold for initiating lipid-lowering therapy. Overall, 88% carried an FH-causing mutation, suggesting that the majority of these children are on their country FH register because of being identified through cascade testing from a mutation positive relative. Few data exits on the future lipid-trajectory in children with a pathogenic FH mutation, but one report [33] showed that 11 of 25 children with an FH mutation and LDL-C below 3.5 mmol/L developed hypercholesterolemia during 3.8 years of follow up. It is possible that children with low LDL-C in our study may have inherited a “milder” mutation, and a detailed analysis of the genotype-phenotype relationships is in preparation.

### 4.1 Strengths and limitations

One of the major strengths of this large dataset is that it allows a snapshot of the way children with FH are currently being treated across different countries in Europe. To do this we created a “minimum data set” and data dictionary of key variables (available on request from authors) which we believe should be helpful in any future cross-country comparisons. The main limitation is that not all countries had routinely been collecting all the data analysed here, and for example collection of the family history of CHD was missing from several countries. For young children, whose parent are likely to be aged only 30–40 years old, a family history of premature CHD in their first degree relatives is very unlikely, and so the definition was expanded to include premature CHD in second degree relatives, for example in grandparents. While this data may be more relevant in making a clinical decision about statin initiation, the accuracy of such data is often hard to verify. However age of onset of premature CHD in relatives is a key factor in the clinical decision as to the age to initiate statin therapy [[9], [10], [11], [12], [13], [14], [15]], and therefore standardisation of this information would be very helpful. Similarly, we requested plasma levels of Lp(a), a well-known CHD risk marker, but this had not been routinely measured across the countries. We did not collect information on dietary differences across countries, as these would have been difficult to standardise, but these are likely to have contributed to some extent to the differences in characteristics seen here. A further limitation is that not all countries used the same procedures on data collection and data monitoring, which is likely to have contributed to the heterogeneity of the data. In particular, the Greek children were all selected as having an identified mutation so overall this group are likely to have a more “severe” FH phenotype than those from other countries. We also did not collect data as to whether the child was an index case or had been identified from cascade testing, and this would be useful information to collect in the future. Finally, because of funding constraints we were not able to make a comprehensive survey of the number of identified FH children in any of the countries and only a single physician in each country was requested to submit the data that they had. We therefore are unable to estimate what proportion of the predicted number of FH children have been identified in each country, but, as with adults with FH [1] it is likely to be extremely low.

### 4.2 Conclusions

Overall, the majority of children with FH in these eight countries are being appropriately managed with regard to age of initiation and dose of statin used. However, there are a sizable proportion, which differs between countries, of children aged over 10 years old who have LDL-C above the EAS guideline recommendation of 3.5 mmol/l, who are not being treated with statin or other lipid lowering medication. Since ultrasound studies have demonstrated significant carotid intima-media thickening in non-treated FH children of this age compared to their non-FH siblings [[34], [35], [36]], and clinical trials have shown that statin treatment can reverse this [19,37,38], considering initiation of statin therapy by the age of 8–10 years is a recommendation in most recent guidelines [[9], [10], [11], [12], [13], [14]]. While, for ethical and practical reasons, there are no long term randomised-placebo controlled trials to examine the benefit of statin initiation at this age and LDL-C level, observational studies over at least 20 years support the reduction of CVD risk associated with this approach [17]. While the proportion of children over the age of 10 years being treated with ezetimibe varies widely across Europe, the vast majority of those taking this medication do achieve the 3.5 mmol/l LDL-C target. While further long-term data from registries such as this would be valuable to confirm the safety and benefit of early statin therapy, working with paediatricians to emphasise the high but avoidable risk of future premature CHD risk in untreated young people with FH, and to develop tools to help clinicians appropriately assess this risk, is therefore a priority.

## Author contributions

Uma Ramaswami is the Clinical Lead for the UK Register and co-wrote the manuscript. Marta Futema carried out the statistical analysis and co-wrote the manuscript. Steve Humphries is project lead for the Paediatric Register, devised the analytical strategy and co-wrote the manuscript. All co-authors commented on drafts and approved the final version of the manuscript. Susanne Greber-Platzer (Medical University of Vienna, Austria) recruited most of the Austrian FH-affected children into the national registry.

## Financial support

The European Register is supported by a grant from the International Atherosclerosis Society (Pfizer number 24052829). The UK register is supported by funds from the British Heart Foundation (BHF); HEART UK, Cardiac Network Co-ordinating Group Wales and the Royal College of Physicians. SEH is a BHF Professor and is funded by PG08/008, and by the National Institute for Health Research University College London Hospitals Biomedical Research Centre. MF is funded by the Fondation Leducq Transatlantic Networks of Excellence Program grant (no. 14 CVD03). MV and TF are funded by the Ministry of Health of the Czech Republic (grant nr. 15-28277A). The Austrian FH register has been supported by funds from the Austrian Heart Foundation and the Tyrolean Regional Government. The study sponsors had no role in study design, the collection, analysis, and interpretation of data, the writing of the report or the decision to submit the manuscript for publication. No honorarium, grant, or other form of payment was given to anyone to produce the manuscript.

## Declaration of competing interest

The authors declared they do not have anything to disclose regarding conflict of interest with respect to this manuscript.
