# Supplementary material for: Comparison of the characteristics at diagnosis and treatment of children with heterozygous familial hypercholesterolaemia (FH) from eight European countries
Source: Atherosclerosis. 2020 Jan;292:178–87. doi: 10.1016/j.atherosclerosis.2019.11.012 (PMC6949888; doi:10.1016/j.atherosclerosis.2019.11.012)
Supplement: Multimedia component 1 [file mmc1.docx]

**Supplementary Data for manuscript:**

**Comparison of the characteristics at diagnosis and treatment of children with Heterozygous Familial Hypercholesterolaemia (FH) from eight European countries**

Uma Ramaswami MD^1*^, Marta Futema^2*^, Martin P. Bogsrud^3,4^, Kirsten B. Holven^3,5^, Jeanine Roeters van Lennep^6^, Albert Wiegman^7^, Olivier S. Descamps^8^, Michal Vrablik^9^, Tomas Freiberger^10^, Hans Dieplinger^11^, Susanne Greber-Platzer^12^, Gabriele Hanauer-Mader^13^, Mafalda Bourbon^14^ , Euridiki Drogari^15^, Steve E Humphries^16^

^1^ Lysosomal Disorders Unit, Royal Free Hospital, London.

^2^ Centre for Heart Muscle Disease, Institute for Cardiovascular Science, University College London, London.

^3^ National Advisory Unit on Familial Hypercholesterolemia, Department of Endocrinology, Morbid Obesity and Preventive Medicine, Oslo University Hospital, Oslo, Norway.

^4^ Unit for Cardiac and Cardiovascular Genetics, Department of Medical Genetics, Oslo University Hospital, Oslo, Norway.

^5^ Department of Nutrition, University of Oslo, Oslo, Norway.

^6^ Departments of Cardiology and Internal Medicine, Erasmus Medical Center, Rotterdam, The Netherlands.

^7^ Department of Pediatrics and Academic Medical Center, Amsterdam, The Netherlands.

^8^ Centres Hospitaliers Jolimont, Lipid Clinic, Haine-Saint-Paul, Belgium.

^9^ Third Department of Internal Medicine, General University Hospital and First Faculty of Medicine, Charles University, U Nemocnice 1, 128 08 Prague 2, Czech Republic.

^10^ Centre for Cardiovascular Surgery and Transplantation, Pekarska 53, 656 91 Brno, Czech Republic, and Medical Faculty, Masaryk University, Brno, Czech Republic.

^11^ Institute of Genetic Epidemiology, Department of Genetics and Pharmacology, Medical University of Innsbruck, Schöpfstraße 41, 6020 Innsbruck, Austria.

^12^ Division of Pediatric Pulmonology, Allergology and Endocrinology, Department of Pediatrics and Adolescent Medicine, Medical University Vienna.

^13^ FH Registry of the Austrian Atherosclerosis Society, Vienna, Austria.

^14^ Cardiovascular Research Group, Research and Development Unit, Department of Health Promotion and Chronic Diseases, National Institute of Health Doutor Ricardo Jorge, Lisbon, Portugal and University of Lisboa, Faculty of Sciences, BioISI - Biosystems & Integrative Sciences Institute, Lisboa, Portugal.

^15^ First Department of Pediatrics, National and Kapodistrian University of Athens and Department of Inborn Errors of Metabolism and Inherited Dyslipidemias, “MITERA” Children’s Hospital, Athens, Greece.

^16^ Centre for Cardiovascular Genetics, Institute for Cardiovascular Science, University College London, London.

*These authors contributed equally to this manuscript

**Corresponding author:** Professor Steve E Humphries, Centre for Cardiovascular Genetics, , Institute of Cardiovascular Science, 5 University Street, University College London, London, UK WC1E 6JF, Tel 0207 679 6962 , email: [steve.humphries@ucl.ac.uk](mailto:steve.humphries@ucl.ac.uk)

**Fig S1.** Box plot (median and quartiles) of baseline LDL-C (mmol/L) for each country cohort of FH children. The mean baseline LDL-C for all cohorts is shown as the red dashed line (5.7 mmol/L). Outliers are indicated as individual dots.


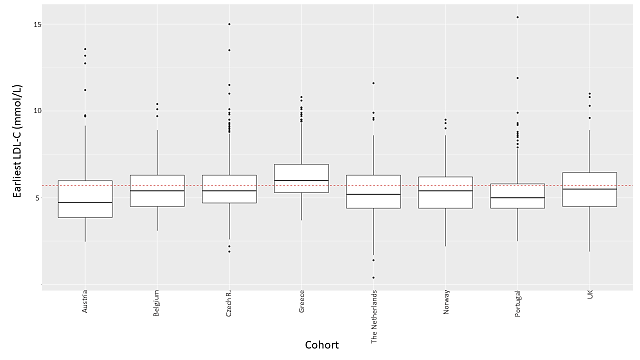


**Table S1.** Selection criteria used in the different countries

| **Country** | **Diagnostic Criteria** | **Time period of collection** | **Estimated Proportion by CT** | **Relevant publications** |
| --- | --- | --- | --- | --- |
| Norway | Genetic testing* | 2014-2016 | 100% | (1) |
| UK | SB | 2014-2014 | >90% | (2) |
| The Netherlands | DLCN | 1993-2018 | >90% | (3, 4) |
| Belgium | DLCN | 2014-2018 | >90 | (5) |
| Czech Republic | MedPed | 1998-2018 | ~50% |  |
| Austria | SB | 2015-2018 | ~75% | (6) |
| Portugal | SB | 1999-2018 | ~17% | (7) |
| Greece | SB | 1993-2018 | 100% |  |

SB = Simon Broome, DLCN = Dutch Lipid Clinic Network Score, CT = Cascade Testing

*plus children with LDL-C levels >4.9mmol/l and a first or second degree relative with an FH mutation, were included

**Table S2.** Overall mean (+SD) characteristics of children with baseline LDL-C less than and more than 4.0mmol/l

|  |  | **Total LDL-C**  **< 4.0mmol/l**  **n =235 (7.7%)** | **Total LDL-C**  **> 4.0mmol/l**  **N =2762 (92.3%)** | **P value** |
| --- | --- | --- | --- | --- |
| **Age at diagnosis** | median (IQR) | 10 (7) | 7 (8) | 3.5x10^-14^ |
| **Number of boys (%)** |  | 114 (49) | 1330 (48) | NS |
| **Number with family history of CHD (%)** | data available for 192 and 1541 children^1^ | 36 (18.9) | 223 (14.5) | 0.06 |
| **Number (%) with mutation^2^** | data available for 214 and 2674 | 188 (87.9) | 2388 (89.3) | NS |
| **Baseline lipids (mmol/l):** mean (SD) | | | | |
| **Total Cholesterol** |  | 5.19 (0.75) | 7.78 (1.34) | <2x10^-16^ |
| **LDL-Cholesterol** |  | 3.33 (0.55) | 5.90 (1.30) | <2x10^-16^ |
| **HDL-Cholesterol** |  | 1.46 (0.39) | 1.44 (0.35) | NS |
| **Triglyceride** |  | 0.88 (0.48) | 0.95 (0.51) | 0.05 |
| **Number receiving statins (%)** | data available for 235 and 2715 children | 53 (23) | 1630 (59) | <2x10^-16^ |

^1^excluding Greece and Norway where data were not collected. Calculated only on those with available information .

^2^ % was calculated only on children who actually had DNA test done

**Table S3.** Frequencies of FH genes where mutations were identified in each country cohort.

The values are presented as numbers and (%). NA=mutation carrier but the mutation details not available.

| **Country** | **Mutated gene** | | | |  |
| --- | --- | --- | --- | --- | --- |
|  | ***LDLR*** | ***APOB*** | ***PCSK9*** | **NA** | **Total** |
| Norway | 231 (93) | 12 (5) | 5 (2) | 0 | 248 |
| UK | 173 (94) | 11 (6) | 0 | 0 | 184 |
| The Netherlands | 302 (93) | 24 (7) | 0 | 0 | 326 |
| Belgium | 112 (93) | 5 (4) | 3 (3) | 0 | 120 |
| Czech Republic | 315 (61) | 201 (39) | 0 | 3 | 519 |
| Austria | 36 (75) | 5 (10) | 1 (2) | 6 | 48 |
| Portugal | 162 (91) | 14 (8) | 2 (1) | 0 | 178 |
| Greece | 1000 (100) | 0 | 0 | 0 | 1000 |
| **Overall** | 2343 (89) | 272 (10) | 11 (0.4) | 9 | 2623 |

**Table S4.** LDL-C-lowering treatment by country. Taking all treatments together there were 1789 individuals who received some medication (hence the majority of Ezetimibe is given in combination with statins). ^a^there were 6 individuals where the statin type was not recorded. NA=not available

**Table S5.** Comparison of children older than 10 years who are on statins and either do or do not receive Ezetimibe.

**Table S6.** Number of children receiving statins by follow-up age.

**Table S7.** Characteristics of children receiving or not receiving statin treatment (not selected by age group).

^a^ In the majority of countries the treated children are older than those who are not treated, but the overall comparison suggests the opposite. This result is due to the very young Greek cohort of 1000 children.

|  | **Country** | **On statin treatment (n=1702)** | **Not on statin treatment (n=1315)** | ***p* value** |
| --- | --- | --- | --- | --- |
| **N (%) by treatment** | Norway | 145 (58) | 105 (42) |  |
|  | UK | 134 (45) | 164 (55) |  |
|  | The Netherlands | 253 (74) | 90 (26) |  |
|  | Belgium | 117 (68) | 54 (32) |  |
|  | Czech Republic | 98 (16) | 502 (84) |  |
|  | Austria | 44 (69) | 20 (31) |  |
|  | Portugal | 77 (26) | 214 (74) |  |
|  | Greece | 834 (83) | 166 (17) | **< 2.2x10^-16^** |
| **N (%) males** | Norway | 74 (51) | 48 (46) | NS |
|  | UK | 68 (51) | 85 (52) | NS |
|  | The Netherlands | 112 (44) | 50 (56) | NS |
|  | Belgium | 52 (44) | 20 (37) | NS |
|  | Czech Republic | 54 (55) | 220 (44) | 0.05 |
|  | Austria | 22 (50) | 8 (40) | NS |
|  | Portugal | 43 (56) | 88 (41) | 0.03 |
|  | Greece | 422 (51) | 83 (50) | NS |
|  | **All** | 847 (50) | 602 (46) | **0.02** |
| **N (%) with an identified mutation** | Norway | 145 (100) | 103 (98) | NS |
|  | UK | 84 (63) | 100 (61) | NS |
|  | The Netherlands | 246 (97) | 80 (89) | 2.1x10^-5^ |
|  | Belgium | 96 (82) | 24 (44) | 0.001 |
|  | Czech Republic | 83 (85) | 391 (78) | NS |
|  | Austria | 39 (89) | 9 (45) | 0.01 |
|  | Portugal | 50 (65) | 128 (60) | NS |
|  | Greece | 834 (100) | 166 (100) | NS |
|  | **All** | 1577 (93) | 1001 (76) | **< 2.2x10^-16^** |
| **N (%) with family history of CHD (1st degree relative)** | Norway | NA | NA | NA |
|  | UK | 32 (24) | 30 (18) | NS |
|  | The Netherlands | 43 (17) | 13 (14) | NS |
|  | Belgium | 30 (26) | 8 (15) | NS |
|  | Czech Republic | 8 (8) | 38 (8) | NS |
|  | Austria | 11 (25) | 6 (30) | NS |
|  | Portugal | 16 (21) | 28 (13) | NS |
|  | Greece | NA | NA | NA |
|  | **All** | 140 (20) | 123 (12) | **1.9x10^-5^** |
| **Median (IQR) age at diagnosis (years)** | Norway | 9 (4) | 7 (5) | 1.3x10^-5^ |
|  | UK | 11 (4) | 9 (6) | 0.0007 |
|  | The Netherlands | 11 (5) | 9 (7) | 0.0008 |
|  | Belgium | 11 (6) | 11 (7) | NS |
|  | Czech Republic | 9 (6) | 10 (6) | NS |
|  | Austria | 8 (8) | 7 (5) | NS |
|  | Portugal | 11 (4) | 10 (6) | NS |
|  | Greece | 3 (1) | 2 (1) | 4.1x10^-12^ |
|  | **All** | 4 (8) | 9 (7) | **< 2.2x10^-16a^** |
| **First recorded TC (mmol/l)** | Norway | 7.73 (1.26) | 6.60 (1.29) | 7.6x10^-11^ |
|  | UK | 7.80 (1.49) | 7.16 (1.47) | 0.0003 |
|  | The Netherlands | 7.32 (1.34) | 6.11 (1.81) | 1.4x10^-7^ |
|  | Belgium | 7.78 (1.45) | 6.61 (1.20) | 1.7x10^-7^ |
|  | Czech Republic | 8.25 (1.56) | 7.24 (1.37) | 2.9x10^-8^ |
|  | Austria | 7.28 (1.78) | 5.62 (0.94) | 8.9x10^-6^ |
|  | Portugal | 7.55 (1.57) | 7.12 (1.54) | 4.1x10^-2^ |
|  | Greece | 8.16 (1.20) | 7.99 (1.27) | NS |
|  | **All** | 7.89 (1.37) | 7.13 (1.49) | **< 2.2x10^-16^** |
| **First recorded LDL-C (mmol/l)** | Norway | 5.80 (1.21) | 4.72 (1.24) | 6.3x10^-11^ |
|  | UK | 5.89 (1.49) | 5.22 (1.43) | 0.0002 |
|  | The Netherlands | 5.57 (1.30) | 4.46 (1.74) | 5.1x10^-7^ |
|  | Belgium | 5.87 (1.39) | 4.73 (1.13) | 8.0x10^-8^ |
|  | Czech Republic | 6.42 (1.51) | 5.38 (1.32) | 3.4x10^-9^ |
|  | Austria | 5.36 (1.64) | 3.79 (0.85) | 4.8x10^-6^ |
|  | Portugal | 5.66 (1.52) | 5.16 (1.42) | 0.02 |
|  | Greece | 6.22 (1.25) | 6.14 (1.26) | NS |
|  | **All** | 6.01 (1.35) | 5.26 (1.43) | **< 2.2x10^-16^** |
| **First recorded HDL-C (mmol/l)** | Norway | 1.42 (0.37) | 1.52 (0.34) | 0.03 |
|  | UK | 1.38 (0.30) | 1.42 (0.35) | NS |
|  | The Netherlands | 1.36 (0.44) | 1.29 (0.35) | NS |
|  | Belgium | 1.43 (0.40) | 1.44 (0.33) | NS |
|  | Czech Republic | 1.34 (0.30) | 1.41 (0.41) | NS |
|  | Austria | 1.41 (0.36) | 1.35 (0.33) | NS |
|  | Portugal | 1.46 (0.33) | 1.46 (0.42) | NS |
|  | Greece | 1.52 (0.29) | 1.46 (0.30) | 0.01 |
|  | **All** | 1.46 (0.34) | 1.42 (0.38) | **0.02** |
| **First recorded TG (mmol/l)** | Norway | 0.95 (0.55) | 0.90 (0.37) | NS |
|  | UK | 1.07 (0.56) | 1.03 (0.53) | NS |
|  | The Netherlands | 0.99 (0.48) | 1.01 (0.57) | NS |
|  | Belgium | 1.13 (0.69) | 0.90 (0.50) | 0.02 |
|  | Czech Republic | 1.07 (0.54) | 1.01 (0.67) | NS |
|  | Austria | 0.95 (0.54) | 0.94 (0.35) | NS |
|  | Portugal | 0.98 (0.53) | 1.00 (0.56) | NS |
|  | Greece | 0.83 (0.40) | 0.83 (0.31) | NS |
|  | **All** | 0.93 (0.49) | 0.97 (0.53) | **0.008** |

**Table S8.** Overall comparison between girls and boys from all cohorts. Numbers in brackets represent %, interquartile range or standard deviation. Lipids are shown in mmol/L. NS= not significant

**Table S9.** Characteristics of children older than 10 years receiving or not receiving statin treatment.

|  | **Country** | **On statin treatment (n=1424)** | **Not on statin treatment (n=352)** | ***p* value** |
| --- | --- | --- | --- | --- |
| **N (%) by treatment** | Norway | 144 (63) | 83 (37) |  |
|  | UK | 123 (56) | 97 (44) |  |
|  | The Netherlands | 234 (85) | 41 (15) |  |
|  | Belgium | 97 (75) | 32 (25) |  |
|  | Czech Republic | 95 (71) | 38 (29) |  |
|  | Austria | 38 (79) | 10 (21) |  |
|  | Portugal | 74 (61) | 47 (39) |  |
|  | Greece | 619 (99) | 4 (1) | < 2.2x10^-16^ |
| **N (%) males** | Norway | 73 (51) | 34 (41) | NS |
|  | UK | 63 (51) | 46 (47) | NS |
|  | The Netherlands | 102 (44) | 22 (54) | NS |
|  | Belgium | 40 (41) | 10 (31) | NS |
|  | Czech Republic | 54 (57) | 12 (32) | 0.01 |
|  | Austria | 19 (50) | 3 (30) | NS |
|  | Portugal | 41 (55) | 18 (38) | NS |
|  | Greece | 320 (52) | 2 (50) | NS |
|  | **All** | 705 (50) | 144 (42) | 0.01 |
| **N (%) with an identified mutation** | Norway | 144 (100) | 82 (99) | NS |
|  | UK | 75 (61) | 52 (54) | NS |
|  | The Netherlands | 231 (99) | 38 (93) | 0.007 |
|  | Belgium | 81 (84) | 16 (50) | 0.02 |
|  | Czech Republic | 80 (84) | 27 (71) | 0.03 |
|  | Austria | 33 (87) | 4 (40) | NS |
|  | Portugal | 48 (65) | 22 (47) | NS |
|  | Greece | 619 (100) | 4 (100) | NS |
|  | **All** | 1292 (92) | 241 (70) | < 2.2x10^-16^ |
| **N (%) with family history of CHD (1st degree relative)** | Norway | NA | NA | NA |
|  | UK | 31 (25) | 17 (18) | NS |
|  | The Netherlands | 41 (18) | 8 (20) | NS |
|  | Belgium | 27 (28) | 3 (9) | NS |
|  | Czech Republic | 8 (8) | 1 (3) | NS |
|  | Austria | 10 (26) | 1 (10) | NS |
|  | Portugal | 14 (19) | 7 (15) | NS |
|  | Greece | NA | NA | NA |
|  | **All** | 127 (20) | 36 (14) | 1.9x10^-5^ |
| **Median (IQR) age at diagnosis (years)** | Norway | 9 (4) | 8 (4) | 0.008 |
|  | UK | 11 (4) | 11 (5) | NS |
|  | The Netherlands | 11 (5) | 11 (7) | NS |
|  | Belgium | 12 (4) | 13 (4) | NS |
|  | Czech Republic | 9 (6) | 10 (4) | NS |
|  | Austria | 8 (11) | 10 (4) | NS |
|  | Portugal | 11 (4) | 12 (5) | NS |
|  | Greece | 3 (1) | 3 (0) | NS |
|  | **All** | 6 (8) | 11 (5) | < 2.2x10^-16^ |
| **First recorded TC (mmol/l)** | Norway | 7.72 (1.26) | 6.66 (1.29) | 1.0x10^-8^ |
|  | UK | 7.79 (1.50) | 7.02 (1.45) | 0.0002 |
|  | The Netherlands | 7.31 (1.33) | 5.91 (1.43) | 3.1x10^-7^ |
|  | Belgium | 7.85 (1.47) | 7.00 (1.25) | 0.002 |
|  | Czech Republic | 8.23 (1.58) | 7.47 (1.22) | 0.004 |
|  | Austria | 7.29 (1.81) | 5.84 (0.71) | 0.0003 |
|  | Portugal | 7.58 (1.59) | 6.70 (0.91) | 0.0002 |
|  | Greece | 8.20 (1.22) | 6.05 (0.17) | 2.6x10^-6^ |
|  | **All** | 7.89 (1.39) | 6.76 (1.35) | < 2.2x10^-16^ |
| **First recorded LDL-C (mmol/l)** | Norway | 5.80 (1.22) | 4.80 (1.27) | 2.6x10^-8^ |
|  | UK | 5.85 (1.51) | 5.04 (1.36) | 6.6x10^-5^ |
|  | The Netherlands | 5.56 (1.27) | 4.27 (1.40) | 1.1x10^-6^ |
|  | Belgium | 5.94 (1.43) | 4.97 (1.24) | 5.2x10^-4^ |
|  | Czech Republic | 6.41 (1.53) | 5.52 (0.99) | 1.5x10^-4^ |
|  | Austria | 5.36 (1.68) | 3.95 (0.54) | 7.5x10^-5^ |
|  | Portugal | 5.67 (1.54) | 4.75 (0.80) | 6.3x10^-5^ |
|  | Greece | 6.25 (1.27) | 4.05 (0.29) | 0.0002 |
|  | **All** | 5.99 (1.39) | 4.85 (1.26) | < 2.2x10^-16^ |
| **First recorded HDL-C (mmol/l)** | Norway | 1.42 (0.37) | 1.50 (0.33) | NS |
|  | UK | 1.40 (0.30) | 1.42 (0.34) | NS |
|  | The Netherlands | 1.37 (0.45) | 1.29 (0.35) | NS |
|  | Belgium | 1.45 (0.39) | 1.54 (0.33) | NS |
|  | Czech Republic | 1.34 (0.30) | 1.36 (0.49) | NS |
|  | Austria | 1.40 (0.35) | 1.38 (0.35) | NS |
|  | Portugal | 1.46 (0.33) | 1.54 (0.52) | NS |
|  | Greece | 1.52 (0.27) | 1.68 (0.09) | 0.05 |
|  | **All** | 1.45 (0.34) | 1.45 (0.39) | NS |
| **First recorded TG (mmol/l)** | Norway | 0.95 (0.55) | 0.91 (0.37) | NS |
|  | UK | 1.06 (0.57) | 1.07 (0.54) | NS |
|  | The Netherlands | 0.99 (0.48) | 1.04 (0.76) | NS |
|  | Belgium | 1.12 (0.7) | 1.02 (0.57) | NS |
|  | Czech Republic | 1.06 (0.54) | 1.29 (0.74) | NS |
|  | Austria | 0.99 (0.61) | 1.04 (0.37) | NS |
|  | Portugal | 0.98 (0.54) | 1.00 (0.61) | NS |
|  | Greece | 0.84 (0.42) | 0.62 (0.19) | NS |
|  | **All** | 0.94 (0.51) | 1.04 (0.59) | 0.009 |
| **Latest recorded LDL-C (mmol/l)** | Norway | 3.60 (1.22) | 4.49 (1.29) | 8.7x10^-7^ |
|  | UK | 3.97 (1.35) | 4.77 (1.32) | 3.8x10^-5^ |
|  | The Netherlands | 3.68 (1.26) | 3.75 (1.30) | NS |
|  | Belgium | 3.72 (1.35) | 4.50 (1.33) | 0.006 |
|  | Czech Republic | 3.59 (1.20) | 4.20 (1.44) | 0.03 |
|  | Austria | 3.54 (1.33) | 3.32 (0.50) | NS |
|  | Portugal | 3.83 (1.28) | 3.90 (1.05) | NS |
|  | Greece | 2.57 (0.35) | 2.68 (0.13) | NS |
|  | **All** | 3.20 (1.12) | 4.32 (1.33) | < 2.2x10^-16^ |
| **N (%) with LDL-C>3.5 mmol/l at latest visit** | Norway | 59 (41) | 64 (77) | <0.0001 |
|  | UK | 61 (56) | 77 (87) | <0.0001 |
|  | The Netherlands | 101 (44) | 19 (49) | <0.0001 |
|  | Belgium | 50 (52) | 22 (69) | <0.0001 |
|  | Czech Republic | 42 (46) | 22 (63) | <0.0001 |
|  | Austria | 16 (42) | 3 (30) | NS |
|  | Portugal | 38 (52) | 26 (58) | <0.0001 |
|  | Greece | 5 (0.8) | 0 (0) | NS |
|  | **All** | 372 (23) | 233 (66) | <0.0001 |

References:

1. Bogsrud MP, Langslet G, Wium C, Johansen D, Svilaas A, Holven KB. Treatment goal attainment in children with familial hypercholesterolemia: A cohort study of 302 children in Norway. J Clin Lipidol. 2018;12(2):375-82.

2. Ramaswami U, Cooper J, Humphries SE, Group FHPRS. The UK Paediatric Familial Hypercholesterolaemia Register: preliminary data. Arch Dis Child. 2017;102(3):255-60.

3. Galema-Boers JM, Versmissen J, Roeters van Lennep HW, Dusault-Wijkstra JE, Williams M, Roeters van Lennep JE. Cascade screening of familial hypercholesterolemia must go on. Atherosclerosis. 2015;242(2):415-7.

4. Narverud I, van Lennep JR, Christensen JJ, Versmissen J, Gran JM, Iversen PO, et al. Maternal inheritance does not predict cholesterol levels in children with familial hypercholesterolemia. Atherosclerosis. 2015;243(1):155-60.

5. Wiegman A, Gidding SS, Watts GF, Chapman MJ, Ginsberg HN, Cuchel M, et al. Familial hypercholesterolaemia in children and adolescents: gaining decades of life by optimizing detection and treatment. Eur Heart J. 2015;36(36):2425-37.

6. Kreissl A, Walleczek N, Espina PR, Hallwirth U, Greber-Platzer S. Selective screening for familial hypercholesterolemia in Austrian children - first year results. BMC Pediatr. 2019;19(1):208.

7. Medeiros AM, Alves AC, Bourbon M. Mutational analysis of a cohort with clinical diagnosis of familial hypercholesterolemia: considerations for genetic diagnosis improvement. Genetics in medicine : official journal of the American College of Medical Genetics. 2016;18(4):316-24.
